# Amplitude of Low-Frequency Oscillations in Major Depressive Disorder With Childhood Trauma

**DOI:** 10.3389/fpsyt.2020.596337

**Published:** 2021-01-22

**Authors:** Zhuoying Wu, Qianyi Luo, Huawang Wu, Zhiyao Wu, Yingjun Zheng, Yuling Yang, Jianfei He, Yi Ding, Rongjun Yu, Hongjun Peng

**Affiliations:** ^1^Department of Clinical Psychology, The Affiliated Brain Hospital of Guangzhou Medical University, Guangzhou, China; ^2^Department of Psychiatry, The Affiliated Brain Hospital of Guangzhou Medical University, Guangzhou, China; ^3^Department of Management, Hong Kong Baptist University, Hong Kong, China

**Keywords:** major depressive disorder, childhood trauma, low-frequency fluctuation, resting state, MRI, insula, primary somatosensory cortex, dorsal anterior cingulate cortex

## Abstract

Major Depressive Disorder (MDD) with childhood trauma is one of the functional subtypes of depression. Frequency-dependent changes in the amplitude of low-frequency fluctuations (ALFF) have been reported in MDD patients. However, there are few studies on ALFF about MDD with childhood trauma. Resting-state functional magnetic resonance imaging was used to measure the ALFF in 69 MDD patients with childhood trauma (28.7 ± 9.6 years) and 30 healthy subjects (28.12 ± 4.41 years). Two frequency bands (slow-5: 0.010–0.027 Hz; slow-4: 0.027–0.073 Hz) were analyzed. Compared with controls, the MDD with childhood trauma had decreased ALFF in left S1 (Primary somatosensory cortex), and increased ALFF in left insula. More importantly, significant group × frequency interactions were found in right dorsal anterior cingulate cortex (dACC). Our finding may provide insights into the pathophysiology of MDD with childhood trauma.

## Introduction

Major depressive disorder (MDD) is described as long sadness and interest loss in generally pleasant activities, following with inability to perform daily activities, over a period of at least 2 weeks (1). At present, depending on its characteristics, MDD can be divided into different subtypes (2).

Childhood trauma is considered as a key factor affecting the occurrence of depression (3, 4). Some scholars believe that MDD with childhood trauma may be one of the functional subtypes of depression (5). To gain an insight of MDD and its heterogeneity, latest studies have been focusing on its clinically relevant depression hypotypes (6). Previous studies have found that nearly half of depression patients have experienced childhood trauma (7, 8), and their clinical characteristics are different from those of ordinary depression patients (9). For instance, symptoms of MDD are often more serious. The symptoms of MDD include higher suicide risk, serious cognitive impairment, and decreased response to antidepressants (10). In addition, there are some specific changes in brain imaging of depression patients with childhood trauma. Brain network studies reported that there are constant disruptions of resting-state networks (RSNs) in patients with MDD, including increased connection between the DMN and FPN, hyperconnectivity in the default mode network (DMN), and hyporconnectivity in the frontoparietal network (FPN) (11). Saleh et al. reported that in depressive subjects, greater early life stress exposure was linked to slower processing speeds and smaller orbitofrontal cortex volumes; however, in non-depressive subjects, it was associated with faster speeds and larger volumes (12). Some studies have found that, compared with the healthy control subjects, amplitude of low-frequency fluctuations (ALFF) in bilateral amygdala and left orbit/cerebellum is increased, while fALFF in the left inferior temporal gyrus and right middle frontal gyrus is decreased in children with MDD after trauma. On MDD, early life stress was positively correlated with regional activity in the left cerebellum and the right posterior central/infratemporal/superior frontal cingulate gyrus and inferior frontal gyrus (13).

Since typical task-based functional magnetic resonance imaging (fMRI) scans can filter out low-frequency physiological noise and keep the high-frequency component of the signal it may lead to confusion about performance differences or activity patterns of specific tasks between healthy subjects and patients. Resting-state fMRI (rs-FMRI), which is applied to evaluate interactions between brain regions when a subject is not performing a specific task (14), has been widely applied to identify the neural correlation to psychiatric disorders. Low frequency fluctuation amplitude analysis (ALFF) measures voxel-by-voxel fluctuations in the amplitude of a blood oxygen level related (BOLD) signal at very low frequencies (typically 0.010–0.080 Hz) (15), which are pointed out to reflect autonomous brain activity (16). Signals in different frequency bands may lead to distinguish brain oscillations with physiological functions and specific characteristics (17). Entire low-frequency spectrum is usually subdivided into four bands (slow-2, 0.198–0.250 Hz; slow-3, 0.073–0.198 Hz; slow-4, 0.027–0.073 Hz; slow-5, 0.010–0.027 Hz) (18). The slow-4 and slow-5 bands are the most associated to gray matter (GM) signals, which are helpful for identifying associations between the functional processing and disease (19). More recent studies have examined ALFF at slow-4 (0.027–0.073 Hz) and slow-5 (0.010–0.027 Hz).

Although ALFF analyses are widely used to study depression, few ALFF studies on MDD have specifically examined MDD with childhood trauma. Therefore, more research is needed to examine the pathophysiology and etiology of MDD with childhood trauma, to help with the early diagnosis and treatment of this subtype of depression, and further to enhance patients' quality of life. The search for biological markers of disease mechanism, treatment choices, diagnosis, or prognosis has become one of the principal goals of depression' neuroimaging (11). Our research aims to identify potential biomarkers to differentiate individuals with MDD with childhood trauma from individuals with ordinary depression.

## Materials and Methods

### Subjects

Ninety-nine subjects were recruited for this study. Among them, 69 were unmedicated MDD patients with childhood trauma and 30 were non-depressed comparison subjects (HC). Sixty-nine unmedicated subjects with MDD ranging from 18 to 45 were recruited from the outpatient and inpatient units The Affiliated Brain Hospital of Guangzhou Medical University. The absence or presence of MDD was evaluated by the structured clinical interview (SCID) for DSM-V diagnostic criteria. Patients with other neurological disorders, psychiatric axis-I or axis-II disorders, electroconvulsive therapy, medication used in the past 6 months, clinically relevant abnormalities in laboratory examinations or their medical history, and contraindication for magnetic resonance imaging (MRI) were excluded. Depression severity were evaluated by Zung's Self-Rating Depression Scale (SDS) and The 17-item Hamilton Depressive Rating Scale (HAMD) (20). The Childhood Truama Questinnaire (CTQ-SF) was used to measure childhood adversity. The CTQ-SF consist of five subscales: sexual abuse (SA), emotional abuse (EA), physical abuse (PA), emotional neglect (EN) and physical neglect (PN) (21). The cut off points for subscales are: SA grade ≥ 8, EA grade ≥ 13, PA grade ≥ 10, PN grade ≥ 10, and EN grade ≥ 15 (8, 22). In addition, 30 gender- age-, and education-matched healthy subjects were recruited from the community near the hospital. Before enrollment, all participants were fully gave notice of the information of the research, and written informed consent was formal obtained. Researches were executed under the Declaration of Helsinki and ratified by The Affiliated Brain Hospital of Guangzhou Medical University Ethics Committee.

### MRI Data Acquisition

Philips 3T MR systems (Philips, Best, The Netherlands) were used to acquire imaging data. Subjects were asked to wear earplugs and positioned in the coil for support. Participants were requested to lie still with their eyes closed but to remain awake, and all participants reported that they did not fall asleep during the whole experiment. A total of 180 volumes of echo planar images were obtained axially (repetition time, 3,000 ms; echo time, 30 ms; slices, 36; thickness, 4 mm, no slice gap; field of view, 240 × 240 mm^2^; resolution, 64 × 64; flip angle, 90°).

### Image Pre-processing

The first 10 volumes of each functional time series for the instability of the initial MRI signal and the adaptation of participants to the circumstance were discarded. The fMRI images were sliced acquisition corrected, head-motion corrected, normalized to the standard SPM5 Montreal Neurological Institute (MNI) template, and then re-sampled to 3-mm cubic voxels. Data were filtered using typical temporal bandpass (0.010–0.080 Hz), slow-4 bandpass (0.027–0.073 Hz), and slow-5 bandpass (0.010–0.027 Hz) separately after linear detrending. REST software was used to calculate ALFF (by Dr. Yong He, http://resting-fmri.sourceforge.net). The filtered time series was transformed into a frequency domain with a fast Fourier transform (FFT) (parameters: taper percent = 0, FFT length = shortest), and the power spectrum was then obtained. Since the power of a given frequency is proportional to the square of the amplitude of this frequency component of the original time series in the time domain, the square root was calculated at each frequency of the power spectrum and the averaged square root was obtained across 0.010–0.080 Hz at each voxel. This averaged square root was taken as the ALFF. For standardization purposes, the ALFF of each voxel was divided by the global mean ALFF value.

A corrected threshold of family-wise error (FWE) of *p* < 0.05 at cluster level was set. Coordinates were reported in MNI coordinates, as used by SPM.

### Statistical Analysis

SPSS 17.0 software was used to analysis the general data. Mean ± SD was used to describe the continuous data that conformed the normal distribution. Between the data analysis of MDD with childhood trauma group and health control group, independent sample *t*-test was performed. When calculating the group differences within the ALFF measurement and the correlation between the clinical measurements and ALFF, age, gender, and education level were included as covariates. In depressed subjects, relationship between depression severity and ALFF values were analyzed by Pearson's correlations. Respectively, *post-hoc* test and simple effect analyses were conducted with the mALFF values averaged in order to further investigate the differences in frequency bands and groups. The above results are statistically significant with *P* < 0.05.

## Results

### Demographics

Clinical data and demographic of healthy and depressed subjects are showed in Table 1. The mean age of the 69 patients is 28.7 ± 9.6 years, and the average year of education is 13.27 ± 3.32 years. The illness duration is 21.32 ± 12.08 months. Thirty controls are recruited, of which the average age is 28.12 ± 4.41 years, and the mean years of education is 14.12 ± 3.24. There is no significant difference in gender, education state, age between two groups.

**Table 1:** Demographics and clinical characteristics.

	**HC (*****n*** **=** **30)**		**MDD with childhood trauma (*****n*** **=** **69)**		**T/*P*-value**
	**Mean**	**SD**	**Mean**	**SD**	
Age(y)	28.12	4.41	28.70	9.6	0.23
Gender (male/female) (n)	19/11		39/30		0.42
Education (years)	14.12	3.24	13.27	3.32	0.32
Illness duration (m)	n.a.	n.a.	21.32	12.08	n.a.
HAMD	n.a.	n.a.	28.39	9.42	n.a.
SDS	n.a.	n.a.	52.81	9.26	n.a.

*HC, healthy controls; SD, standard deviation; MDD, major depression disorder; HAMD, Hamilton Depressive Rating Scale; SDS, Self-Rating Depression scale*.

### Correlations Between Clinical Data and the ALFF

In insula, a highly positive correlation between HAMD total score and the ALFF (*p* < 0.01) was found. On the contrary, a strong negative correlation between depressive symptom severity and ALFF was detected in the S1 and dACC (*p* < 0.01) (Table 2).

**Table 2:** Brain regions showing statistically significant correlation between ALFF and depression severity in terms of HAMD scores in subjects with MDD with CT.

**Brain regions**	**Brodmann areas**	**Cluster size**	**MNI coordinates**			***R*-values**
			**x**	**y**	**z**	
Anterior Insula	13	87	−39	9	0	+0.54
S1	3a, 3b, 1, and 2	44	9	−42	75	−0.66
dACC	32	155	12	−6	39	−0.73

*BA, Brodmann area; MNI, Montreal Neurologic Institute; MDD, major depressive disorder; S1, primary somatosensory cortex; dACC, dorsal Anterior Cingulate Cortex*.

### ALFF Frequency Main Effect

**Figure 1:**
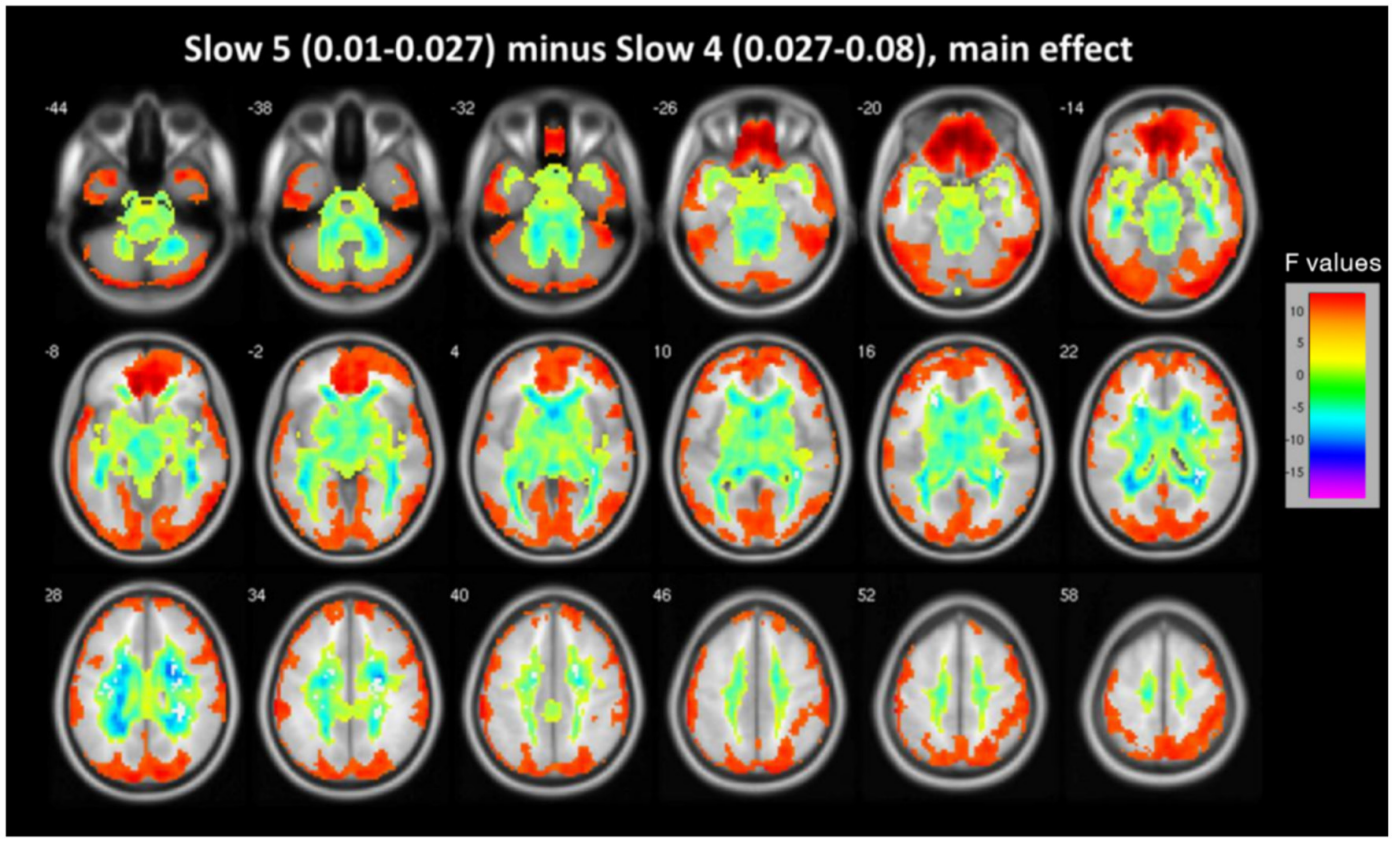
The main effect for frequency band on ALFF. Most of the brain showed marked differences in ALFF between the two frequency bands (slow-4 vs. slow-5). Warm color represents greater ALFF in the slow-5 band than in the slow-4 band, while cool color represents lower ALFF. A two-way repeated measures ANOVA obtained the results.

Main effects from the two-way repeated measure ANOVA are shown in Figure 1. In midbrain, basal ganglia, cingulate cortex and fusiform gyrus, ALFF of slow 4 is obviously larger than that of slow 5, while ALFF of slow 5 is larger in lingual gyrus, middle temporal gyrus, inferior frontal gyrus and medial frontal ventral gyrus (see Figure 1).

### ALFF Group Main Effect

**Figure 2:**
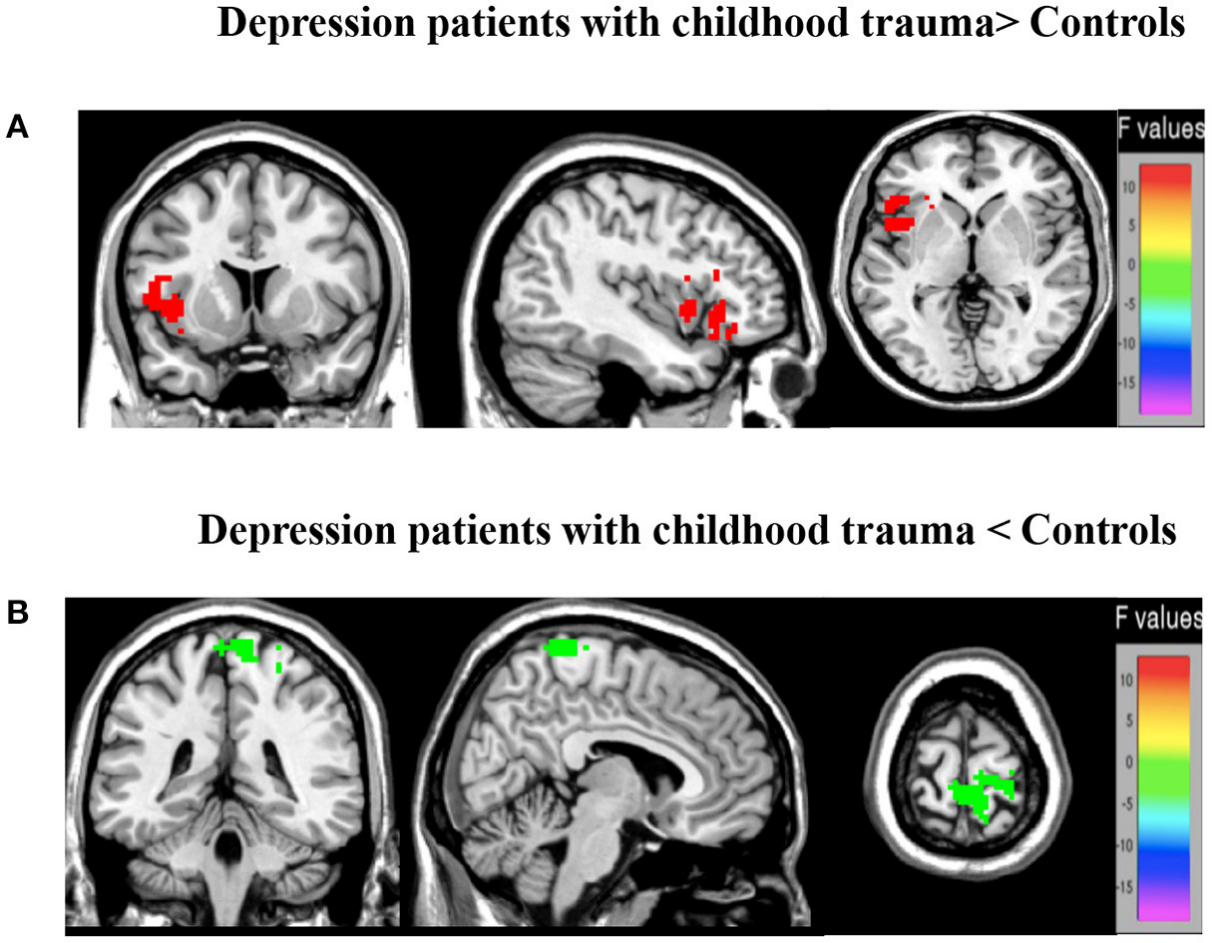
Brain regions showing marked group main effect **(A,B)** on ALFF. **(A)** Warm colors represent reduced ALFF in the MDD with CT when compared with HC; **(B)** cool colors represent the decreased ALFF in the young depression group when compared with HC.

Differences between groups are demonstrated in Table 3 and Figure 2. Compared to controls, patients exhibited decreased ALFF in a cluster in the postcentral gyrus (sensorimotor cortex), [*x* = 9, −42, 75], *Z* = 5.72, voxels = 154, PFWE < 0.05, see Figure 2A. Patients had higher ALFF in the left anterior insula, [−39, 9, 0], *Z* = 3.29, voxels = 22, PFWE < 0.05 (Figure 2B).

**Table 3:** The brain region showing marked group main effect and interaction effect of group and frequency (slow−4 and slow−5) on ALFF.

	**Anatomical**	**Left/**	**Size**	**Peak level P**	**MNI(mm)**			**Voxel**
	**region**	**right**	**(voxels)**	**(FWE-corrected)**	**x**	**y**	**z**	***Z*-value**
Depression-control	Anterior insula	L	22	0.042	−39	9	0	3.29
Control-depression	S1	L	154	0.021	9	−42	75	5.72
Group × Frequency interaction	dACC	R	56	0.017	12	−6	39	5.20

*dACC, dorsal Anterior Cingulate Cortex; S1, primary somatosensory cortex*.

### Group X Frequency Interaction

**Figure 3:**
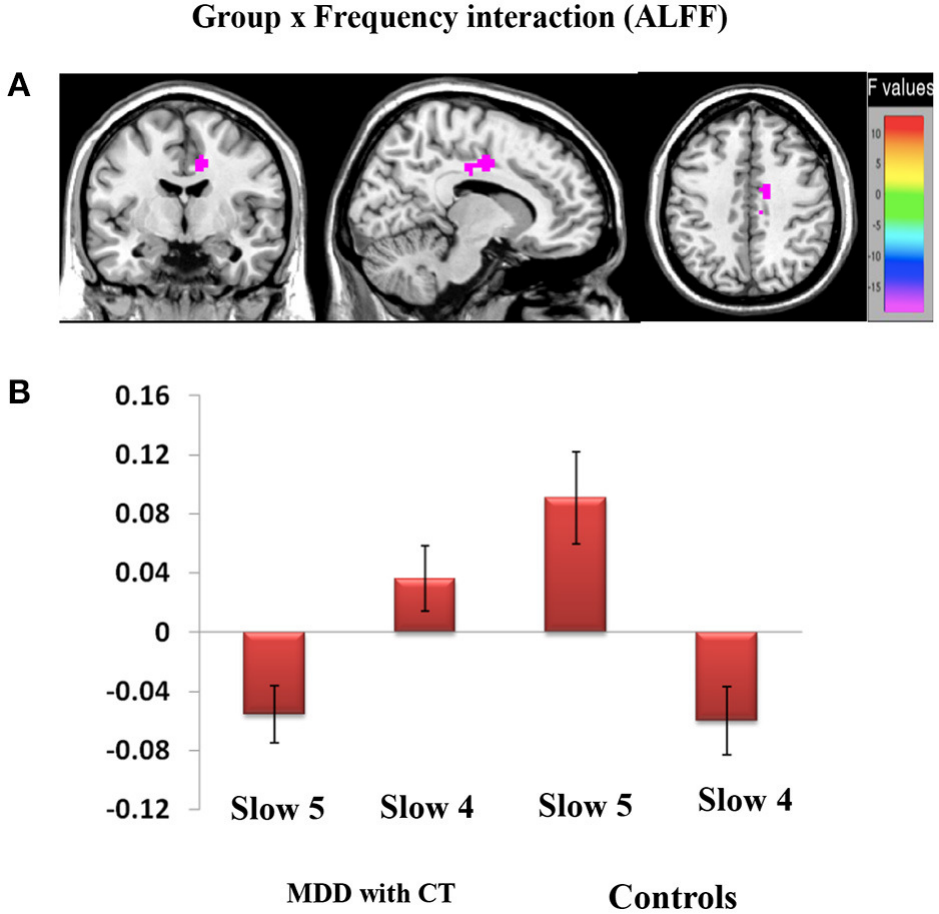
Brain regions showing significant group and frequency (slow-4 and slow-5) interaction **(A,B)** effects on ALFF. **(A)** The interaction between frequency band and group on ALFF. A two-way repeated-measure ANOVA and *post-hoc* test obtained the results. The beta values for dACC were plotted **(B)** to show the directions of the interaction.

We found the significant group by frequency interaction in dACC, [12, −6, 39], *Z* = 5.20, voxels = 56, PFWE < 0.05, corrected at cluster-level see Figure 3A. The interaction is plotted in Figure 3.

## Discussion

Functional MRI has been **broadly** used to explore the brain systems's dysfunction in patients with psychiatric disorders. Our study found that the frequency-dependent changes in the ALFF occur in MDD patients with childhood trauma. ALFF analysis is applied to examine the amplitude of low-frequency oscillations (LFOs) in MDD with childhood trauma in different frequency bands, namely slow-5 and slow-4.

Our study is the first systematic investigation to explore frequency-dependent changes in LFO amplitudes (as indexed by the ALFF) from resting-state fMRI signals in MDD patients with childhood trauma. In different brain regions, the main frequency-dependent effects varies according to the different frequency bands: slow-4 oscillations were greater than slow-5 oscillations primarily in subcortical regions, such as midbrain, basal ganglia, cingulate cortex, and fusiform gyrus; and slow-5 oscillations were greater than slow-4 oscillations in several cortical areas, such as the inferior frontal gyrus, ventromedial frontal gyrus and middle temporal gyrus. Compared with controls, major depressive patients with childhood trauma indicated decreased ALFF values in S1 and increased ALFF values in insula. Moreover, a remarkable interaction was identified between frequency bands and groups in dACC, which suggests that the observed dynamic changes in low-frequency fluctuations may be related to frequency-dependent. Due to this reason, we decided to analyze group differences in the ALFF across the slow-5 and slow-4 ranges.

### Group Differences in ALFF Between Frequency Bands

Previous studies of the ALFF have also detected the differences between brain areas in the slow-4 and slow-5 bands (18, 23, 24). Although, from different frequency bands, the significance and source of signals are not clear, it has been suggested that the different low-frequency bands relate to different neural processes and physiological functions (17, 25). Lower frequency oscillations have been linked with long-range connections and the integration of large neuronal systems, while signals from higher frequency bands have been linked to more local neuronal assemblies and short-range connections (25). Therefore, regions of the cerebral cortex such as the inferior frontal gyrus, ventromedial frontal gyrusand, and middle temporal gyrus, may facilitate long-distance connectivity in many large networks. Conversely, subcortical regions such as fusiform gyrus, cingulate cortex, and basal ganglia may mainly contribute to fast local events, which are regulated by extensive slow oscillations (25, 26). Additionally, compared with the slow-4 band, we found higher ALFF values in the slow-5 band in most of the main subregions of the default mode network (DMN) (27), including inferior frontal gyrus, middle temporal gyrus and ventromedial frontal gyrus. Our results are in accord with the previous studies on MDD patients which suggested that the slow-5 band shows stronger LFOs and higher functional activity in the majority of DMN subregions (18, 28, 29). It seems that differences in ALFF between frequency bands in MDD with childhood trauma are similar to MDD patients. Interestingly, in the cingulate cortex, lower ALFF were found in the slow-5 band, which differ from the results of ALFF studies into other diseases (28, 29). The cingulate cortex, a key DMN region shows a strong connectivity in primates with the parahippocampal gyrus and entorhinal cortex, and thus with the hippocampal memory system (30). It has also been reported that the functional connectivity of the cingulate cortex is related to rumination in depression (31). We assume that MDD patients with childhood trauma suffer mainly from a dysfunction of the cingulate cortex.

### Increased ALFF Activity in Insular in MDD With CT Patients

The insula is regarded as a hub for external and internal information exchange (32). Accumulating evidence are based on studying changes in insula function in MDD patients, abnormalities in the insula caused by MDD, and dysregulation of the interaction between the insula and other important functional brain networks (such as DMN and central executive network) show can be explored (33–35). The insula plays an important role in generating and processing emotional information, including feelings of anxiety and disgust (36, 37). In short, previous findings demonstrate that the insula plays an important role in the pathogenesis of MDD patients with childhood trauma. Previous neuroimaging studies have found that the abnormal function of the insula is tightly related to the symptoms of depression, including reports that resting-state brain functional connectivity in insula can more accurately distinguish healthy controls from patients with depression (38). Numerous studies have shown a close relationship between changes in insula function and depression, but the results of these studies are inconsistent. Su et al. found that the ALFF decreases in the insula of depressive patients (39, 40), but some scholars found that the ALFF was increased in the insula of depressive patients (41). Whether MDD patients experience childhood trauma may be one of the reasons leading to the inconsistent. In our study, we found that the ALFF in the insula of the depressive group is observably higher than that in the HC group, indicating the dysfunction of insular in MDD patients with childhood trauma. This dysfunction may be the key factor that severely impairs the ability of MDD patients with childhood trauma to regulate their emotions. Our results show that compared to HC, MDD patients have an increased ALFF in the insula, which indicates abnormal insula activities, and it may further lead to a declined in the ability of regulating the negative emotions.

### Decreased ALFF Activity in S1 in MDD With CT Patients

S1, parallel to the central sulcus, is located in the postcentral gyrus (42). S1 is responsible for integrating pain-related information. In animal models, researchers have made a solid conclusion that S1 is involved in sensory-discriminative pain processing; that is, it encodes the location and intensity of pain stimulation (43–45). A number of studies have found that empathizing with others' pain can also activate S1 (46). Moreover, research has shown that the cognitive processing of pain usually begins with activation in prefrontal brain regions, which then further regulates activity in pain-related regions of S1 (47–49). It indicates that the painful somatic symptoms of MDD may relate to functional changes in S1. Several studies have found that when subjects experience different emotions, the activity in S1 also changes and it is highly activated when the subjects focus on their own emotions (50). The result has indicated that S1 takes part in the processing of emotional experience, primarily in the generation of somatosensory emotions. More and more research is supporting the conclusion that S1 is involved in modulating and controlling associatively learned behaviors (51, 52). Schaefer et al. even suggested that S1 functions as an “embodied mind,” which supports the existence of the unconscious self in concert with the embodied aspect of the self (53). In short, these findings suggest that S1 is likely to be an important structure closely related to cognitive function. MDD patients with childhood trauma typically have many symptoms related to cognitive impairment, which may be associated with S1 dysfunction. Previous studies have found an increased GM volume in right S1 in patients with comorbid anxiety and MDD (54), while the total area of the somatosensory cortex is reduced in adolescent patients with MDD and in patients with bipolar disorder (55, 56). Kang et al. reported MDD patient's abnormalities in S1–thalamic functional connectivity related to the typical clinical symptoms of MDD, which indicates that such changes are neurobiological characteristics and potential biomarkers of MDD with childhood trauma (57). However, there has still been no research to report the relationship between changes in functional signals in S1 and the development of MDD. In our study, we found that compared to the healthy controls, MDD with CT patients had a lower ALFF in S1, indicating a lower level of spontaneous neural activity. This implies that the dysfunction in left S1 seems to be related to the development of MDD and lead to a possible decline or loss of sensation. We assume that a lower ALFF in S1 may be a specific signal of MDD with the subtype of childhood trauma.

### Group × Frequency Interaction in dACC

In this study, a significant group × frequency interaction effect on the ALFF value in dACC is observed. This result indicates that the difference in resting brain function may result from an interaction between frequency bands and disease states. Moreover, it suggests that pathological conditions may increase the influence of some particular frequency bands in the brain, especially in dACC, and that each frequency band may have a specific pathological significance. The dACC is connected with areas of the central executive network and the salience network, which are involved in target maintenance and the integration of sensorimotor and visuomotor inputs, and therefore, guides the output of corresponding behaviors (58). Many studies have shown that functional changes in dACC are involved in the pathophysiological process of MDD (59, 60). In addition, some studies have shown that the components of emotional experience related to pain are processed by the anterior insula and dACC (61), and dACC mainly encodes the psychological aspect of pain sensation (62). These conclusions demonstrate that dACC plays a key role in dealing with social pain and functions as a negative information processing system. A number of animal studies have showed that the dACC was activated when infant mammals were separated from their mothers; however, after the damage to dACC, animal infants reduced their whining during separation. This result has indicated that the brain activities that take part in processing pain-related emotional components are closely related to the degree of painful feelings caused by negative social events.

Researchers also investigated whether different forms of negative social events will have the same effect on activating the brain areas that involved in processing painful components of emotion. In conclusion, in the tasks of social evaluation and social loss, brain areas involved in processing painful emotional components were activated (63). The tasks of negative emotional experiences (such as fear, anxiety, anger, disgust, and sadness) and physiological pain both can lead to the increased activity in insula and dACC. Based on that, a conclusion made is that the damage to the function of dACC may lead to an imbalance in negative information processing in MDD with childhood trauma, activating brain areas related to painful emotional components and producing various negative emotions.

We assume that the disease state and spontaneous brain activity may inhibit individuals' functions to each other, thus causing dysfunction in dACC. The remarkable correlation observed between ALFF in the right dACC within slow-5 suggests that the LFO amplitude in the region may be applied to monitor the disease progress of MDD with childhood trauma.

### Limitations

Our study used a cross-sectional design with a relatively small sample. Other confounding differences between the two groups, such as social economic status and mood, may also explain our findings. Future studies may further investigate how ALFF changes as the depression progresses. Importantly, it would be interesting to directly compare MDD with childhood trauma with MDD without such trauma to isolate the effect of childhood trauma on brain networks in MDD. The exact origins and mechanisms underlying ALFF remain elusive. More studies are needed to comprehend the functional significance of ALFF changes in MDD.

## Conclusions

In conclusion, our results show that changes in the ALFF occur in various brain regions in patients suffering MDD with CT, which indicates that the subtypes of MDD involve the dysfunction of multiple areas of the brain. The CT results of MDD patients demonstrate abnormal LFO amplitudes in many brain regions, consist of subcortical regions, cortical areas, insula, and S1, which highlight abnormalities in LFO amplitudes in MDD with CT and provide insights into understanding the pathophysiology of major depressive disorder.

## Data Availability Statement

The raw data supporting the conclusions of this article will be made available by the authors, without undue reservation.

## Ethics Statement

The studies involving human participants were reviewed and approved by Ethics Committee of Affiliated Brain Hospital of Guangzhou Medical University. Written informed consent to participate in this study was provided by the participants' legal guardian/next of kin.

## Author Contributions

ZW and QL designed the study and drafted the primary manuscript. HW, ZW, and YZ supervised the recruitment and made statistical analyses. YY, JH, and YD took part in recruitment and data management. RY and HP made further revisions of the manuscript. All the authors had read and approved the final manuscript.

## Conflict of Interest

The authors declare that the research was conducted in the absence of any commercial or financial relationships that could be construed as a potential conflict of interest.

## Abbreviations

ALFF: amplitude of low-frequency fluctuations
BOLD: blood oxygenation level dependent
DMN: Default-mode network
CTQ-SF: Childhood Truama Questinnaire
dACC: dorsal anterior cingulate cortex
FC: functional connectivity
FWE: family-wise error
FFT: fast Fourier transforms
fMRI: functional magnetic resonance imaging
fALFF: fractional amplitude of low frequency fluctuations
GM: gray matter
HC: healthy controls
HAMD: Hamilton Depressive Rating Scale
LFO: low-frequency oscillations
MDD: Major Depressive Disorder
MRI: magnetic resonance imaging
MPFC: medial prefrontal cortex
MNI: Montreal Neurological Institute
MTG: middle temporal gyrus
S1: primary somatosensory cortex
SCID: structured clinical interview
STD: subthreshold depression
SDS: Zung's Self-Rating Depression Scale
TRD: treatment-resistant depression.

## References

[1] Park LT Zarate CA Depression in the primary care setting. N Engl J Med. (2019) 380:559–68. 10.1056/NEJMcp1712493 30726688 PMC6727965

[2] Alexopoulos GS. Depression in the elderly. Lancet. (2005) 365:1961–70. 10.1016/S0140-6736(05)66665-2 15936426

[3] Heim C Newport DJ Mletzko T Miller AH Nemeroff CB. The link between childhood trauma and depression: insights from HPA axis studies in humans. Psychoneuroendocrinology. (2008) 33:693–710. 10.1016/j.psyneuen.2008.03.008 18602762

[4] Scheuer S Wiggert N Brückl TM Awaloff Y Uhr M Lucae S. Childhood abuse and depression in adulthood: the mediating role of allostatic load. Psychoneuroendocrinology. (2018) 94:134–42. 10.1016/j.psyneuen.2018.04.020 29775876

[5] Heim C Mletzko T Purselle D Musselman DL Nemeroff CB. The dexamethasone/corticotropin-releasing factor test in men with major depression: role of childhood trauma. Biol Psychiatr. (2008) 63:398–405. 10.1016/j.biopsych.2007.07.002 17825799

[6] Ulbricht CM Chrysanthopoulou SA Levin L Lapane KL. The use of latent class analysis for identifying subtypes of depression: a systematic review. Psychiatr Res. (2018) 266:228–46. 10.1016/j.psychres.2018.03.003 29605104 PMC6345275

[7] Nelson J Klumparendt A Doebler P Ehring T. Childhood maltreatment and characteristics of adult depression: meta-analysis. Br J Psychiatr. (2017) 210:96–104. 10.1192/bjp.bp.115.180752 27908895

[8] Xie P Wu K Zheng Y Guo Y Yang Y He J. Prevalence of childhood trauma and correlations between childhood trauma, suicidal ideation, and social support in patients with depression, bipolar disorder, and schizophrenia in southern China. J Affect Disord. (2018) 228:241–8. 10.1016/j.jad.2017.11.011 29223913

[9] Wiersma JE Hovens JG van Oppen P Giltay EJ van Schaik DJ Beekman AT. The importance of childhood trauma and childhood life events for chronicity of depression in adults. J Clin Psychiatr. (2009) 70:983–9. 10.4088/jcp.08m04521 19653975

[10] Gamble SA Talbot NL Duberstein PR Conner KR Franus N Beckman AM. Childhood sexual abuse and depressive symptom severity: the role of neuroticism. J Nerv Ment Dis. (2006) 194:382–5. 10.1097/01.nmd.0000218058.96252.ac 16699389

[11] Yu M Shinohara RT Oathes DJ Cook PA Duprat R Moore TM. Childhood trauma history is linked to abnormal brain connectivity in major depression. Proc Natl Acad Sci USA. (2019) 116:8582–90. 10.1073/pnas.1900801116 30962366 PMC6486762

[12] Saleh A Potter GG McQuoid DR Boyd B Turner R MacFall JR. Effects of early life stress on depression, cognitive performance and brain morphology. Psychol Med. (2017) 47:171–81. 10.1017/S0033291716002403 27682320 PMC5195852

[13] Du L Wang J Meng B Yong N Yang X Huang Q. Early life stress affects limited regional brain activity in depression. Sci Rep. (2016) 6:25338. 10.1038/srep25338 27138376 PMC4853783

[14] Arnone D McIntosh AM Ebmeier KP Munafò MR Anderson IM. Magnetic resonance imaging studies in unipolar depression: systematic review and meta-regression analyses. Eur Neuropsychopharmacol. (2012) 22:1–16. 10.1016/j.euroneuro.2011.05.003 21723712

[15] Zhou Y Wang K Liu Y Song M Song SW Jiang T. Spontaneous brain activity observed with functional magnetic resonance imaging as a potential biomarker in neuropsychiatric disorders. Cognitive Neurodyn. (2010) 4:275–94. 10.1007/s11571-010-9126-9 22132039 PMC2974101

[16] Fox MD Raichle ME. Spontaneous fluctuations in brain activity observed with functional magnetic resonance imaging. Nat Rev Neurosci. (2007) 8:700–11. 10.1038/nrn2201 17704812

[17] Knyazev GG. Motivation, emotion, and their inhibitory control mirrored in brain oscillations. Neurosci Biobehav Rev. (2007) 31:377–95. 10.1016/j.neubiorev.2006.10.004 17145079

[18] Wang L Kong Q Li K Su Y Zeng Y Zhang Q. Frequency-dependent changes in amplitude of low-frequency oscillations in depression: a resting-state fMRI study. Neurosci. Lett. (2016) 614:105–11. 10.1016/j.neulet.2016.01.012 26797652

[19] Zuo XN Di Martino A Kelly C Shehzad ZE Gee DG Klein DF. The oscillating brain: complex and reliable. Neuroimage. (2010) 49:1432–45. 10.1016/j.neuroimage.2009.09.037 19782143 PMC2856476

[20] Helmreich I Wagner S Mergl R Allgaier A-K Hautzinger M Henkel V Sensitivity to changes during antidepressant treatment: a comparison of unidimensional subscales of the Inventory of Depressive Symptomatology (IDS-C) and the Hamilton Depression Rating Scale (HAMD) in patients with mild major, minor or subsyndromal depression. Eur Arch Psychiatr Clin Neurosci. (2011) 262:291–304. 10.1007/s00406-011-0263-x 21959915

[21] Bernstein DP Stein JA Newcomb MD Walker E Pogge D Ahluvalia T. Development and validation of a brief screening version of the childhood trauma questionnaire. Child Abuse Negl. (2003) 27:169–90. 10.1016/s0145-2134(02)00541-0 12615092

[22] Jansen K Cardoso TA Fries GR Branco JC Silva RA Kauer-Sant'Anna M. Childhood trauma, family history, and their association with mood disorders in early adulthood. Acta Psychiatr Scand. (2016) 134:281–136. 10.1111/acps.12551 26826334

[23] Yu R Chien Y-L Wang H-LS Liu C-M Liu C-C Hwang T-J. Frequency-specific alternations in the amplitude of low-frequency fluctuations in schizophrenia. Human Brain Mapp. (2014) 35:627–37. 10.1002/hbm.22203 23125131 PMC6869729

[24] Yang L Yan Y Li Y Hu X Lu J Chan P. Frequency-dependent changes in fractional amplitude of low-frequency oscillations in Alzheimer's disease: a resting-state fMRI study. Brain Imag Behav. (2019) 14:2187–201. 10.1007/s11682-019-00169-6 31478145

[25] Buzs aki G Draguhn A. Neuronal oscillations in cortical networks. Science. (2004) 304:1926–9. 10.1126/science.1099745 15218136

[26] Salvador R Suckling J Schwarzbauer C Bullmore E. Undirected graphs of frequency-dependent functional connectivity in whole brain networks. Philos Trans R Soc B: Biol Sci. (2005) 360:937–46. 10.1098/rstb.2005.1645 16087438 PMC1854928

[27] Raichle ME MacLeod AM Snyder AZ Powers WJ A default mode of brain function. PNAS. (2001) 98:676–82. 10.1073/pnas.98.2.676 11209064 PMC14647

[28] Liu X Wang S Zhang X Wang Z Tian X He Y. Abnormal amplitude of low-frequency fluctuations of intrinsic brain activity in Alzheimer's disease. J Alzheimers Dis. (2014) 40:387–97. 10.3233/JAD-131322 24473186

[29] Wei L Duan X Zheng C Wang S Gao Q Zhang Z. Specific frequency bands of amplitude low-frequency oscillation encodes personality. Hum Brain Mapp. (2014) 35:331–9. 10.1002/hbm.22176 22987723 PMC6869309

[30] Bubb EJ Kinnavane L Aggleton JP. Hippocampal–diencephalic–cingulate networks for memory and emotion: An anatomical guide. Brain Neurosci Adv. (2017) 1:2398212817723443. 10.1177/2398212817723443 28944298 PMC5608081

[31] Berman MG Peltier S Nee DE Kross E Deldin PJ Jonides J. Depression, rumination and the default network. Soc Cogn Affect Neurosci. (2011) 6:548–55. 10.1093/scan/nsq080 20855296 PMC3190207

[32] Mufson EJ Mesulam MM. Insula of the old world monkey. II: Afferent cortical input and comments on the claustrum. J Comp Neurol. (1982) 212:223–37. 10.1002/cne.902120103 7174906

[33] Menon V. Large-scale brain networks and psychopathology: a unifying triple network model. Trends Cogn Sci. (2011) 15:483–506. 10.1016/j.tics.2011.08.003 21908230

[34] Manoliu A Meng C Brandl F Doll A Tahmasian M Scherr M. Insular dysfunction within the salience network is associated with severity of symptoms and aberrant inter-network connectivity in major depressive disorder. Front Human Neurosci. (2014) 7:930. 10.3389/fnhum.2013.00930 24478665 PMC3896989

[35] Kaiser RH Andrews-Hanna JR Wager TD Pizzagalli DA. Large-scale network dysfunction in major depressive disorder: a meta-analysis of resting-state functional connectivity. JAMA Psychiatr. (2015) 72:603–11. 10.1001/jamapsychiatry.2015.0071 25785575 PMC4456260

[36] Singer T Critchley HD Preuschoff K. A common role of insula in feelings, empathy and uncertainty. Trends Cogn Sci. (2009) 13:334–40. 10.1016/j.tics.2009.05.001 19643659

[37] Lamm C Singer T. The role of anterior insular cortex in social emotions. Brain Struct Funct. (2010) 214:579–91. 10.1007/s00429-010-0251-3 20428887

[38] Ambrosi E Arciniegas DB Madan A Curtis KN Patriquin MA Jorge RE. Insula and amygdala resting-state functional connectivity differentiate bipolar from unipolar depression. Acta Psychiatr Scand. (2017) 136:129–39. 10.1111/acps.12724 28369737 PMC5464981

[39] Fitzgerald PB Laird AR Maller J Daskalakis ZJ. A meta-analytic study of changes in brain activation in depression. Human Brain Mapp. (2008) 29:683–95. 10.1002/hbm.20426 17598168 PMC2873772

[40] Su L Cai Y Xu Y Dutt A Shi S Bramon E. Cerebral metabolism in major depressive disorder: a voxel-based meta-analysis of positron emission tomography studies. BMC Psychiatr. (2014) 14:321. 10.1186/s12888-014-0321-9 25407081 PMC4240898

[41] Liu CH Ma X Song LP Fan J Wang WD Lv XY. Abnormal spontaneous neural activity in the anterior insular and anterior cingulate cortices in anxious depression. Behav Brain Res. (2015) 281:339–47. 10.1016/j.bbr.2014.11.047 25513974

[42] Kaas JH Nelson RJ Sur M Lin CS Merzenich MM. Multiple representations of the body within the primary somatosensory cortex of primates. Science. (1979) 204:521–3. 10.1126/science.107591 107591

[43] Guilbaud G Benoist JM Levante A Gautron M Willer JC. Primary somatosensory cortex in rats with pain-related behaviours due to a peripheral mononeuropathy after moderate ligation of one sciatic nerve: neuronal responsivity to somatic stimulation. Exp Brain Res. (1992) 92:227–45. 10.1007/BF00227967 1337325

[44] Follett KA Dirks B. Characterization of responses of primary somatosensory cerebral cortex neurons to noxious visceral stimulation in the rat. Brain Res. (1994) 656:627–32. 10.1016/0006-8993(94)91362-5 7804842

[45] Canavero S Bonicalzi V. Role of primary somatosensory cortex in the coding of pain. Pain. (2013) 154:1156–8. 10.1016/j.pain.2013.02.032 23590938

[46] Apkarian AV Bushnell MC Treede RD Zubieta JK. Human brain mechanisms of pain perception and regulation in health and disease. Eur J Pain. (2005) 9:463–84. 10.1016/j.ejpain.2004.11.001 15979027

[47] Rainville P Duncan GH Price DD Carrier B Bushnell MC. Pain affect encoded in human anterior cingulate but not somatosensory cortex. Science. (1997) 277:5968–71. 10.1126/science.277.5328.968 9252330

[48] Peyron R Laurent B García-Larrea L. Functional imaging of brain responses to pain. A review and meta-analysis. Neurophysiol Clin. (2000) 30:263–88. 10.1016/s0987-7053(00)00227-6 11126640

[49] Rainville P. Brain mechanisms of pain affect and pain modulation. Curr Opin Neurobiol. (2002) 12:195–204. 10.1016/s0959-4388(02)00313-6 12015237

[50] Damasio AR Grabowski TJ Bechara A Damasio H Ponto LL Parvizi J. Subcortical and cortical brain activity during the feeling of self-generated emotions. Nat Neurosci. (2000) 3:1049–56. 10.1038/79871 11017179

[51] Galvez R Weiss C Weible AP Disterhoft JF. Vibrissa-signaled eyeblink conditioning induces somatosensory cortical plasticity. J Neurosci. (2006) 26:6062–8. 10.1523/JNEUROSCI.5582-05.20 16738249 PMC6675235

[52] Chau LS Davis AS Galvez R. Neocortical synaptic proliferation following forebrain-dependent trace associative learning. Behav Neurosci. (2013) 127:285–92. 10.1037/a0031890 23398434

[53] Schaefer M Northoff G. Who am i: the conscious and the unconscious self. Front Hum Neurosci. (2017) 11:126. 10.3389/fnhum.2017.00126 28367120 PMC5355470

[54] Qi H Ning Y Li J Guo S Chi M Gao M. Gray matter volume abnormalities in depressive patients with and without anxiety disorders. Medicine. (2014) 93:e345. 10.1097/MD.0000000000000345 25546687 PMC4602623

[55] Koutsouleris N Meisenzahl EM Borgwardt S Riecher-Rössler A Frodl T Kambeitz J. Individualized differential diagnosis of schizophrenia and mood disorders using neuroanatomical biomarkers. Brain. (2015) 138:2059–73. 10.1093/brain/awv111 25935725 PMC4572486

[56] Schmaal L Hibar DP Sämann PG Hall GB Baune BT Jahanshad N. Cortical abnormalities in adults and adolescents with major depression based on brain scans from 20 cohorts worldwide in the ENIGMA Major depressive disorder working group. Mol Psychiatr. (2017) 22:900–9. 10.1038/mp.2016.60 27137745 PMC5444023

[57] Kang L Zhang A Sun N Liu P Yang C Li G. Functional connectivity between the thalamus and the primary somatosensory cortex in major depressive disorder: a resting-state fMRI study. BMC Psychiatr. (2018) 18:339. 10.1186/s12888-018-1913-6 30340472 PMC6194586

[58] Seeley WW Menon V Schatzberg AF Keller J Glover GH Kenna H. Dissociable intrinsic connectivity networks for salience processing and executive control. J Neurosci. (2007) 27:2349–56. 10.1523/JNEUROSCI.5587-06.2007 17329432 PMC2680293

[59] Wagner G Köhler S Bär KJ. Treatment associated changes of functional connectivity of midbrain/brainstem nuclei in major depressive disorder. Sci Rep. (2017) 7:8675. 10.1038/s41598-017-09077-5 28819132 PMC5561091

[60] Schwartz J Ordaz SJ Kircanski K Ho TC Davis EG Camacho MC. Resting-state functional connectivity and inflexibility of daily emotions in major depression. J Affect Disord. (2019) 249:226–34. 10.1016/j.jad.2019.01.040 30743019 PMC6446895

[61] Price DD. Psychological and neural mechanisms of the affective dimension of pain. Science. (2000) 288:1769–72. 10.1126/science.288.5472.1769 10846154

[62] Treedea R-D Kenshalo DR Gracelyb RH Jones AK. The cortical representation of pain. Pain. (1999) 79:105–11. 10.1016/s0304-3959(98)00184-5 10068155

[63] Eisenberger NI Inagaki TK Muscatell KA Byrne Haltom KE Leary MR. The neural sociometer: brain mechanisms underlying state self-esteem. J Cogn Neurosci. (2011) 23:3448–55. 10.1162/jocn_a_00027 21452934

